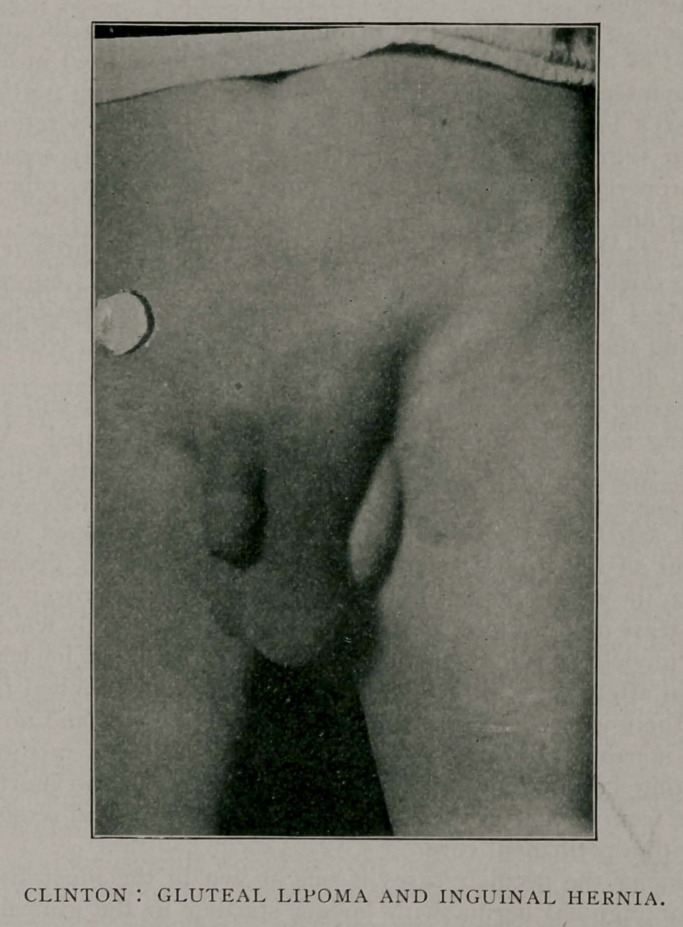# An Analysis of Fifty Herniotomies

**Published:** 1904-09

**Authors:** Marshall Clinton

**Affiliations:** 466 Franklin Street, Buffalo, N. Y., Attending Surgeon, Sisters of Charity, and Erie County Hospitals, Buffalo, N. Y.; Instructor in Surgery in the University of Buffalo


### An Analysis of Fifty Herniotomies.

By MARSHALL CLINTON, M. D., Buffalo, N. Y.,

Attending Surgeon, Sisters of Charity, and Erie County Hospitals, Buffalo, N. Y.; Instructor in Surgery in the University of Buffalo.

During the past few years it has been the author's privilege to operate over fifty hernias of all classes. In this group of cases are several points of interest. The first of the series was a strangulated inguinal hernia, which recurred with great promptitude. In the occasional cases operated during the next three years there are four recurrences; but for the last five years in a careful attempt to closely follow up all cases, I do not find a single recurrence.

A large proportion of the cases in this series were emergency operations due to incarceration or strangulation and the majority were in men over 50. There are in the list five strangulated femoral hernias in women all of whom were over 50 years of age. All recovered and there are no recurrences. The gut was wounded in one case where the tight ring was cut with a sharp

CLINTON: GLUTEAL LIPOMA AND INGUINAL HERNIA.

bistoury used in place of a herniotome, but this accident was repaired and was followed by no bad results.

One fatal case was that of a man 68 years old, in which the entire omentum was incarcerated and inflamed. After resection the patient developed a hyperpyrexia and died in forty-eight hours. Ecchymosis into the scrotum was seen at times but was never troublesome. One internal ring was sewn up too tightly and caused pain by pressure on the cord. One patient was a cryptorchid and the testicle was removed during the operation.

The earliest of my patients were sewn up with silk after the method of Halstead, then in vogue. When infection appeared in the wound the effect of those buried septic silk sutures was such that they ceased to be used. With the betterment of general surgical technic and improvement in the technic of herniotomy, no cases of sepsis were seen and all cases were found healed at the first dressing.

There is some difference of opinion in regard to the proper method of applying sutures and the material to be used in bringing the internal oblique and transversalis to the shelving portion of Poupart's ligament. The whole key to successful operation, as Bassini suggests it, is in getting these layers properly separated and properly approximated without too great tension; otherwise gaping and recurrence is likely to follow.

Where the rings are very large, I have used kangaroo tendon with a double needle, sewing from above downwards; but where the edges approximate with ease I have had no hesitancy in bringing them together with properly prepared catgut, which will absorb in three weeks.

Teasing out the internal oblique and transversalis will aid in bringing together without tension the tissues mentioned. When this is done any absorbable, nonirritating suture that will hold the tissues in place until nature has given a firm scar will be found satisfactory. Occasionally, where the conjoined tendon runs in so far from Poupart's ligament that there is great tension on the structures when drawn together with sutures, it is better to tease out the structures to approximate than to rely wholly on some unyielding suture material. The tissues should lie without tension after the sutures are placed, in every case. When this is done there is no reason for not getting a good result and no need of a nonabsorbable suture material. The modern method of operating herniotomies in uncomplicated cases has no mortality, is free from danger, confines a patient three weeks, and sends him out with a permanent cure.

Among the peculiar cases is one shown in the illustration, a case of inguinal hernia with lipoma starting in the gluteal fold and pushing its way forward until it presented, as shown in the picture. The patient was discharged cured in three weeks.

466 Franklin Street.

---

### EXCORIATED NIPPLES.

℞ Orthoform ................................ 5.0 (75 grs.)
Ether, q. s. ad sol.
Ol. amygd. dulc ............................ 20.0 (5 dr.)

Sig.—Apply locally.

## Figures and Tables

**Figure f1:**